# Transcutaneous Neuromodulation at Posterior Tibial Nerve and ST36 for Chronic Constipation

**DOI:** 10.1155/2014/560802

**Published:** 2014-11-05

**Authors:** Nina Zhang, Zhihui Huang, Feng Xu, Yuemei Xu, Jianfeng Chen, Jieyun Yin, Lin Lin, Jiande D. Z. Chen

**Affiliations:** ^1^Ningbo Pace Translational Medical Research Center, Beilun, Ningbo 210029, China; ^2^Division of Gastroenterology, The First Affiliated Hospital of Nanjing Medical University, 300 Guangzhou Road, Nanjing 210029, China; ^3^Division of Gastroenterology, Sir Run Run Shaw Hospital, School of Medicine, Zhejiang University, Hangzhou 310000, China; ^4^Division of Gastroenterology, Yinzhou Hospital Affiliated to Medical School of Ningbo University, Ningbo 210029, China; ^5^Ningbo Maida Medical Device Inc., Ningbo 210029, China; ^6^Division of Gastroenterology and Hepatology, Johns Hopkins Center for Neurogastroenterology, Baltimore, MD 21224, USA

## Abstract

The aims of this study were to investigate the effects and possible mechanisms of transcutaneous neuromodulation (TN) in patients with chronic constipation. Twelve patients were recruited. The treatment consisted of 2-week TN and 2-week sham-TN which was performed in a crossover design. Bowel habit diary, Patient Assessment of Constipation Symptom (PAC-SYM), Patient Assessment of Constipation Quality of Life (PAC-QOL), and anorectal motility were evaluated. Electrocardiogram was recorded for the assessment of autonomic function during acute TN therapy. It was found that (1) TN improved the frequency of spontaneous defecation. After 2-week TN therapy, 83% patients had more than 3 times bowel movements per week which was significantly different from sham-TN (*P* = 0.01). (2) TN improved PAC-SYM and PAC-QOL scores (*P* < 0.001, resp.). (3) TN significantly decreased the threshold volume to elicit RAIR (*P* < 0.05), ameliorated rectal sensory threshold (*P* = 0.04), and maximum tolerance (*P* = 0.04). (4) TN, but not sham-TN, increased the vagal activity (*P* = 0.01 versus baseline) and decreased the sympathetic activity (*P* = 0.01, versus baseline). It was concluded that needleless TN at posterior tibial nerve and ST36 using a watch-size stimulator is effective in chronic constipation, and the effect was possibly mediated via the autonomic mechanism.

## 1. Introduction

Chronic constipation is a common digestive disease which affects 2% to 26.9% of general population [1]. Chronic constipation is characterized by less frequency of bowel movements (<3 per week), difficult defecation, incomplete evacuation, and other accompanying symptoms [2]. It is considered to impair the quality of life and is associated with psychosocial problems [3]. The annual cost for each patient with chronic constipation is about $7522 in the U.S. [4].

Chronic constipation is known to be classified as slow transit constipation, outlet obstruction, and both. Slow transit constipation is defined as prolonged stool transit (>3 days) through the colon [5]. The outlet obstruction constipation is characterized by impaired relaxation and coordination of abdominal and pelvic floor muscles during evacuation [6]. The regular management for constipation is composed of changes in life style, laxatives, enterokinetic drugs, biofeedback, and surgical procedure. However, the treatment of constipation is still unsatisfactory.

Neuromodulation has recently been shown to be effective for management of chronic constipation [7]. Percutaneous tibial nerve stimulation as a new therapeutic option has been reported to increase stool frequency, decrease the use of laxatives, and improve the quality of life in patients with slow transit constipation [8]. During the past decades, electroacupuncture (EA) has been reported to accelerate gastrointestinal motility both in animals and humans [9–11]. EA at ST36 (Zusanli) was shown to increase gastric emptying in patients with functional dyspepsia [10] and accelerate small bowel transit, colonic contractions, and transit in rats [9, 11]. Little was found in the literature on EA for treating patients with constipation. Although a few studies indicated that EA has the potential for treating constipation [12, 13], these studies were not well designed; either lack of controls or the parameters were unclear which made it difficult to reproduce. Moreover, needle insertion was required in these studies with either tibial nerve stimulation or electroacupuncture, which may generate adverse events such as stress and discomfort around the insertion area, so that may not be acceptable by every patient. In the current study, we hypothesized to use a noninvasive transcutaneous neuromodulation technique, by combining both tibial nerve stimulation and electroacupuncture to evaluate the treatment outcomes in patients with chronic constipation.

Therefore, the aims of this study were to investigate the effects and possible mechanisms of transcutaneous neuromodulation (TN) for the treatment of chronic constipation.

## 2. Materials and Methods

### 2.1. Patients

Adult outpatients, aged 18–75 years, referred to the clinical department at Yinzhou People's Hospital with chronic functional constipation who fulfilled Rome III criteria were enrolled in this study. All the patients had symptoms for at least one year and failed to respond to previous conservative treatment, including laxatives, enemas, and biofeedback. Patients were not recruited into the study if they had any organic diseases causing constipation or neurologic diseases such as multiple sclerosis, rachischisis, Parkinson's disease, and spinal cord injury. Patients who were pregnant or had a history of gastrointestinal surgery except appendectomy or cholecystectomy were excluded. The study protocol was approved by the Ethical Review Committee of Yinzhou People's Hospital. Written consent was obtained from all the patients before the study.

### 2.2. Experimental Protocol

**Figure 1:**
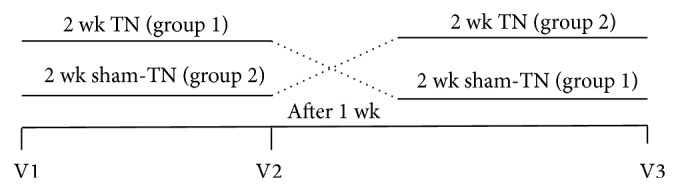
Experimental protocol.

This is a placebo-controlled study (Figure 1). Patients were blinded about the treatment regimens and patients who were familiar with acupoints or meridian were excluded. After one-week run-in period, all of the patients were randomly divided into two groups: one group was allowed to cross over to the sham-TN after receiving 2-week TN therapy followed by 1-week wash-out; the other group was allowed to cross over to the TN therapy after receiving 2-week sham-TN followed with 1-week wash-out. Patients were required for three office visits during the experiments. In each visit, the Patient Assessment of Constipation Symptom (PAC-SYM), the Patient Assessment of Constipation Quality of Life (PAC-QOL) questionnaire, and the anorectal motility test were performed. The patients were asked to fill out the bowel habit diary during TN or sham-TN to record the frequency of defecation, time of defecation, stool quality, and medication for defecation. Lactulose was permitted for use only when the patient cannot tolerate the symptoms. During the last visit, heart rate variability (HRV) was recorded in addition to the symptoms and motility test to evaluate vagal activity.

### 2.3. Transcutaneous Neuromodulation (TN)

Posterior tibial nerve and the acupoint ST36 (Zusanli, either right leg or left leg) were chosen for the TN therapy [8, 14]. Two electrodes were placed for tibial nerve stimulation, one placed at approximately two fingers' breadth up to the malleolus medialis and posterior to the tibia and the other 4 cm above the first electrode. For ST36, one electrode was placed at ST36 and the other was 4 cm below ST36 along the same meridian. The location of sham-TN was applied at nontibial nerve or nonacupoint (not on any meridian). A watch-size digital stimulator (SNM-FDC01, Ningbo Maida Medical Device Inc., Ningbo, China) was used to deliver trains of pulses: train on-time of 2 sec and off-time of 3 sec, pulse width of 0.5 ms, pulse frequency of 25 Hz, and amplitude of 2–10 mA (at the maximum level tolerated by the subject). TN or sham-TN was performed for 1 hour, twice daily.

### 2.4. Symptoms Assessment

The number of spontaneous defecations per week was the major primary outcome. The bowel movements more than 3 times per week were defined as complete response [15]. Time of defecation and stool quality recorded in the bowel diary were documented.

The PAC-SYM was used to assess constipation associated symptoms [16]. The questionnaire included stool symptoms, abdominal symptoms, and rectal symptoms with a total number of 12 items. The higher score ranging from 0 to 4 indicated the higher severity of symptom. The PAC-QOL was a validated questionnaire for the assessment of quality of life in patients with chronic constipation [17]. Four subscales indicated physical discomfort, psychosocial distress, concerns, and satisfaction with a total number of 28 items. The lower score represented the better quality of life.

### 2.5. Anorectal Motility

The anorectal manometry was performed using an eight-channel water perfused system (GAP-08A, Ningbo Maida Medical Device Inc., Ningbo, China) to evaluate (1) threshold volume of rectal distention for eliciting RAIR, (2) anal resting and maximal squeezing pressures, (3) rectal sensory threshold, desire to defecate, urge threshold, and maximum tolerance, (4) increase in rectal pressure during strain, and (5) percentage of paradoxical contractions between the rectum and anal sphincter [18]. Threshold volume of rectal distention for eliciting RAIR was evaluated by rapid inflation of latex balloon in rectum. Rectal sensory threshold, desire to defecate, urge threshold, and maximum tolerance were tested by constant dilation of balloon [19].

### 2.6. Heart Rate Variability (HRV)

HRV test was performed during the last office visit. After 10 minutes of rest, the patients were requested to lie down, and HRV was recorded for 30 min baseline, 30 min with TN/sham-TN and 30 min recovery period (no stimulation). The HRV signal was obtained from the electrocardiogram (ECG) recording. Three ECG electrodes were placed: one on the right manubrium of the sternum, one on the fifth interspace in the left medioclavicular line, and the ground electrode on the right chest. All the electrodes were connected to an ECG amplifier (ECG-201, Ningbo Maida Medical Device Inc., Ningbo, China). The HRV signal was derived using a special program developed and validated in our laboratory by identifying R peaks, calculating R-R intervals, interpolating the R-R intervals so that the time interval between consecutive samples was equal, and finally downsampling the interpolated data to a frequency of 1 Hz [14].

Power spectral analysis was then performed on the segmental HRV data to derive sympathetic and vagal activities. The power in each frequency subband was calculated using a previously validated method [20]. The power in the low frequency band (0.04–0.15 Hz, LF) represents mainly sympathetic activity and the power in the high frequency band (0.15–0.50 Hz, HF) stands purely for parasympathetic or vagal activity. The HF and LF were defined as the area under the curve in the frequency range of 0.15–0.50 Hz and 0.04–0.15 Hz, respectively. The power ratio of LF/HF reflects the balance between the sympathetic and parasympathetic activities.

### 2.7. Statistical Analysis

All data are presented as means ± SE. The paired Student's* t*-test was applied to investigate differences in any of the parameters between baseline and stimulation therapy. It was also used to compare the difference between stimulation therapy and sham stimulation. Chi-square analysis was used to study the difference in the efficacy (rate of complete response of constipation with BM > 3 times/week). Statistical significance was assigned for *P* < 0.05.

## 3. Results

### 3.1. Primary Outcomes

This study was conducted between August 2013 and May 2014. A total of 14 patients (8F, 6M) were enrolled. Two patients were dropped in the middle of the study. The median age of 12 patients (8F, 4M) was 60 years (range 31–75). All patients underwent 2-week TN and 2-week sham-TN of 2 h daily. All bowel diaries were returned and all the questionnaires were completed.

**Figure 2:**
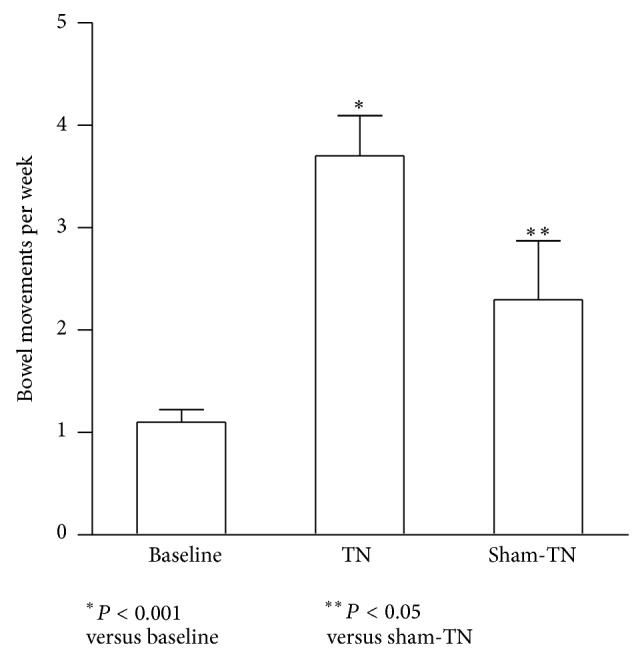
Effects of TN on bowel movement. TN significantly increased weekly bowel movement to over 3 times (*P* < 0.001 versus baseline), which was significantly different from sham-TN treatment (*P* < 0.05).

As shown in the Figure 2, TN significantly improved the frequency of spontaneous defecation after the 2-week treatment. The number of weekly bowel movements was increased from 1.1 ± 0.1 to 3.7 ± 0.4 (*P* < 0.001), which was significantly different from the sham-TN session (2.3 ± 0.6, *P* = 0.01 versus TN). At the end of TN therapy, 10 patients (83%) reported to have more than 3 times bowel movements per week; on the contrary, only 4 patients (33.3%) reported so after the sham-TN treatment (*P* = 0.01; TN versus sham-TN, Chi-square analysis).

TN therapy improved time of defecation. Compared to the baseline, the time of defecation during the TN therapy was decreased from 12.6 ± 0.88 min to 8.2 ± 0.99 min (*P* = 0.02); similar findings were also noted in the sham-TN session; there was no difference between TN and sham-TN. During TN treatment, although not statistically significant, the stool quality showed softer in comparison with baseline (3.2 ± 0.6 versus 1.8 ± 0.8, *P* = 0.11). The percentage of the medication usage recorded in the bowel habit diary was decreased from 50% at baseline to 20% after TN therapy (*P* = 0.03); no significant difference was found between TN and sham-TN (*P* = 0.08).

### 3.2. Secondary Outcomes

**Figure 3:**
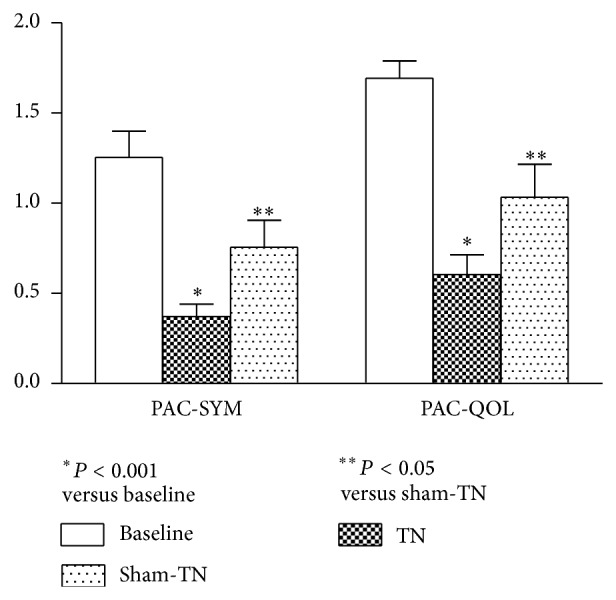
PAC-SYM score and PAC-QOL score before and after TN therapy. TN significantly reduced the PAC-SYM and PAC-QOL scores compared to the baseline (*P* < 0.001, resp.). There was a significant difference between TN and sham-TN treatment (*P* < 0.05).

TN significantly improved the symptoms of constipation assessed by the PAC-SYM questionnaire (Figure 3). After 2-week TN, the PAC-SYM total score was decreased by 70% compared to the baseline (*P* < 0.001). Specifically, the stool symptoms were substantially improved from 2.71 ± 0.21 to 0.95 ± 0.18 after the treatment (*P* < 0.01) and so was the abdominal symptoms (0.75 ± 0.24 versus 0.14 ± 0.19, *P* = 0.04). In addition, TN significantly decreased the symptom scores by 50% compared to the sham-TN treatment (*P* < 0.01).

TN significantly improved the quality of life in patients with constipation (Figure 3). After 2-week TN therapy, the PAC-QOL total score was decreased by 64% compared to the baseline (*P* < 0.001) which was attributed to the amelioration in physical discomfort, concerns, and satisfaction of patients. No difference was noted in the sham-TN treatment compared to the baseline. TN significantly reduced the symptom scores by 42% compared to the sham-TN (*P* < 0.05, Figure 3).

### 3.3. Anorectal Motility

TN significantly improved RAIR and rectal sensation in patients with constipation (Table 1). TN decreased the volume of distention required to achieve internal sphincter relaxation from 41.43 ± 5.94 mL at baseline to 20 ± 4.36 mL (*P* = 0.005), which was significantly different from sham-TN treatment (34.29 ± 4.81, *P* = 0.03, versus TN). Similarly, there were substantial reductions in rectal sensory threshold (40 ± 7.56 at baseline versus 24.29 ± 4.29 at 2 weeks, *P* = 0.04) and maximum tolerance (231.29 ± 19.12 at baseline versus 180 ± 9.0 at 2 weeks, *P* = 0.04). Moreover, the threshold volume was significantly lower with TN treatment than with sham-TN treatment for the first sensation (*P* = 0.02) and maximum tolerance (*P* = 0.03). Neither TN nor sham-TN had effect on the incidence of paradoxical contractions of the anorectum (*P* > 0.05, resp.).

### 3.4. Autonomic Mechanisms of TN

**Figure 4:**
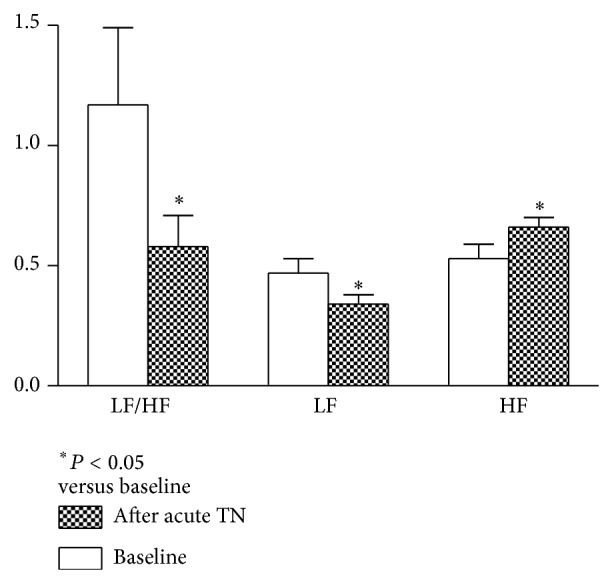
Effects of TN therapy on autonomic activity. TN significantly increased vagal activity and decreased sympathetic activity and sympathovagal balance compared to the baseline (*P* < 0.05). These findings were not noted in sham-TN treatment (*P* < 0.05 versus TN).

TN, but not sham-TN, increased the vagal activity (HF) and decreased the sympathetic activity (LF) (Figure 4). Compared with the baseline, the vagal activity was significantly increased (0.53 ± 0.06 versus 0.66 ± 0.04,  *P* = 0.01) and the sympathetic activity was significantly decreased (0.47 ± 0.06 versus 0.34 ± 0.04,  *P* = 0.01) after acute TN. Similarly, TN markedly decreased the LF/HF ratio from 1.17 ± 0.32 to 0.58 ± 0.13 (*P* < 0.05).

## 4. Discussion

In this study, we developed a novel method of transcutaneous neuromodulation by combining posterior tibial nerve stimulation and EA at ST36 for treating chronic functional constipation. We found that TN significantly increased the frequency of spontaneous defecation and improved the complete response. TN decreased total scores of PAC-SYM and PAC-QOL. In addition, TN ameliorated the rectal distention threshold volume to elicit RAIR and rectal sensation. The improvement of TN on symptoms and anorectal motility may be mediated via the increase of vagal activity and concurrent decrease in sympathetic activity as measured by HRV.

Frequency of application is one of the important factors for response to TN. Transcutaneous electrical stimulation (TES) was used for children with slow transit constipation. Treatment of three times per week for one month did not change the number of bowel movements per week but improved the quality of life [21, 22]. When the frequency of TES was increased to one hour daily, there was a significant increase in episodes of defecation [22]. In this study, TN was performed one hour twice daily, we found that both bowel movement and quality of life were significantly improved with TN treatment, suggesting the importance of treatment frequency, and patients indeed benefit from the daily treatment. The efficacy in our study was comparable to those published in the literatures [23, 24].

In the current study, both acupoint at ST36 and tibial nerve were chosen for TN. It was reported that EA increased the frequency of defecation [25] and decreased stool property, constipation symptom grade, and accompanying symptom grade [26–28], which was consistent with the results of our study. In previous studies, ST36 was also applied to treat constipation by combining it with other acupoints or moxibustion and was shown to be effective [13]. However, in previous studies, EA was performed 2-3 times per week via needles, which was inconvenient and may also generate stress in certain patients. In the current study, TN was a self-administered noninvasive method, no needle insertion was needed, and it can be applied at home. Moreover, we combined both tibial nerve stimulation and EA at ST36 which may maximize the advantage of tibial nerve stimulation and electroacupuncture. In an earlier study, Collins et al. reported that percutaneous tibial nerve stimulation improved symptoms in patients with slow transit constipation [8]. After that, a few studies followed up. In the current study, to the best of our knowledge, this is the first study to use noninvasive TN for the treatment of chronic constipation.

Interestingly, we found that TN significantly decreased the volume of distention required to elicit RAIR in the constipation patients. Previous study reported the impairment of RAIR in patients with constipation; the volume of distention eliciting a relaxation of 50% was higher in patients with constipation compared to the healthy subjects [29]. In the current study, decreased threshold volume with TN may be related to the improvement in symptoms of constipation. Tibial nerve contains fibres arising from both the second and third sacral nerve roots which are the same spinal segments innervating the bladder, rectum, and pelvic floor [8]. In addition, we found that TN at both posterior tibial nerve and ST36 increased parasympathetic activity and decreased sympathetic activity. All these may result in the improvement of RAIR. Further studies are needed to investigate the effects of TN on intrinsic nerves serving internal sphincter [30].

It has been demonstrated that the volume threshold of first sensation was increased in constipated patients [31]. In the current study, TN was found to ameliorate the rectal sensation by reducing the threshold volume of distention on the first sensation and the maximum toleration. This is consistent with the findings reported by other studies [32]. However, whether these physiological changes occur via activation of efferent nerves remain unknown [33].

The effects of the TN therapy on constipation observed in this study might be secondary to changes in autonomic functions. Previous study has reported that EA enhanced the activation of the parasympathetic nervous system and suppressed the activation of sympathetic nervous system [34]. This is in accordance with our results. Reductions in the activities of sympathetic nerves improve the gastrointestinal peristalsis that result in an increase in colonic propagating sequences to speed up transit time [35, 36]. Currently, increasing parasympathetic nerve activities with TN therapy possibly relieve symptoms of constipation through improvement in anorectal physiology or colonic peristalsis.

This is a small size sample pilot study. We did not classify the patient to with or without slow transit, whereas in most of previously published studies, neuromodulation was performed to explore the effects for slow transit constipation [8, 24, 36]. In fact, more than half of all patients with slow transit constipation simultaneously had some degree of outlet obstruction [37, 38]. In the current study, TN did not reduce the percentage of paradoxical contraction but improved RAIR and the rectal sensation. There is need for a further study to explore the effects of TN therapy on colon transit. Another limitation in our study which has to be considered is maintenance of TN. Leong et al. reported 2-month TES held improvement for more than 2 years in one-third of slow transit constipation children [23]. Further studies are needed to investigate whether prolonged TN therapy is required to sustain the effect and how long the efficacy may last after TN treatment.

In conclusion, needleless TN at posterior tibial nerve and ST36 is able to improve the major symptoms of constipation and anorectal motility, especially RAIR and rectal sensation. Home-based noninvasive TN therapy may be a potential treatment for functional constipation.

## Figures and Tables

**Table 1:** Anorectal motility and sensation profiles before and after TN/sham-TN treatment.

	Baseline	After 2-week TN	After 2-week sham-TN
RAIR	41.43 ± 5.94	20 ± 4.36^*^	34.29 ± 4.81^**^
Anal rest pressure (mmHg)	69 ± 1.83	74.84 ± 7.42	75.93 ± 8.59
Maximal squeeze pressure (mmHg)	153.14 ± 24.42	170.56 ± 22.30	150.3 ± 12.20
Duration of anal contraction (s)	12.97 ± 0.42	12.63 ± 0.55	12.8 ± 0.59
First sensation (mL)	40 ± 7.56	24.29 ± 4.29^*^	41.43 ± 7.05^**^
Desire of defecation (mL)	75.71 ± 9.97	60 ± 9.26	85.71 ± 11.52
Urge of defecation (mL)	137.14 ± 17.42	114.29 ± 8.12	148.57 ± 12.99
Maximum tolerable volume (mL)	231.29 ± 19.12	180 ± 9.00^*^	222.86 ± 17.00^**^
Increase in rectal pressure (mmHg)	39.37 ± 7.01	33.94 ± 4.89	36.94 ± 7.41
Paradoxical contraction (%)	90.47 ± 9.53	67.86 ± 17.86	88.1 ± 7.89

^*^ *P* < 0.05 versus baseline; ^**^ *P* < 0.05 versus sham-TN.

## References

[1] Schmidt F. M., Santos V. L. (2014). Prevalence of constipation in the general adult population: an integrative review. *Journal of Wound, Ostomy & Continence Nursing*.

[2] Drossman D. A. (1999). The functional gastrointestinal disorders and the Rome II process. *Gut*.

[3] El-Salhy M., Svensen R., Hatlebakk J. G., Gilja O.-H., Hausken T. (2014). Chronic constipation and treatment options (review). *Molecular Medicine Reports*.

[4] Mason H. J., Serrano-Ikkos E., Kamm M. A. (2002). Psychological state and quality of life in patients having behavioral treatment (Biofeedback) for intractable constipation. *The American Journal of Gastroenterology*.

[5] Gallegos-Orozco J. F., Foxx-Orenstein A. E., Sterler S. M., Stoa J. M. (2012). Chronic constipation in the elderly. *The American Journal of Gastroenterology*.

[6] Foxx-Orenstein A. E., McNally M. A., Odunsi S. T. (2008). Update on constipation: one treatment does not fit all. *Cleveland Clinic Journal of Medicine*.

[7] van Wunnik B. P. W., Baeten C. G. M. I., Southwell B. R. (2011). Neuromodulation for constipation: sacral and transcutaneous stimulation. *Best Practice and Research: Clinical Gastroenterology*.

[8] Collins B., Norton C., Maeda Y. (2012). Percutaneous tibial nerve stimulation for slow transit constipation: a pilot study. *Colorectal Disease*.

[9] Iwa M., Nakade Y., Pappas T. N., Takahashi T. (2006). Electroacupuncture elicits dual effects: stimulation of delayed gastric emptying and inhibition of accelerated colonic transit induced by restraint stress in rats. *Digestive Diseases and Sciences*.

[10] Xu S., Hou X., Zha H., Gao Z., Zhang Y., Chen J. D. (2006). Electroacupuncture accelerates solid gastric emptying and improves dyspeptic symptoms in patients with functional dyspepsia. *Digestive Diseases and Sciences*.

[11] Yin J., Chen J., Chen J. D. Z. (2010). Ameliorating effects and mechanisms of electroacupuncture on gastric dysrhythmia, delayed emptying, and impaired accommodation in diabetic rats. *The American Journal of Physiology—Gastrointestinal and Liver Physiology*.

[12] Xu J., Jia C.-S., Qin L., Xu X.-K. (2012). Comparative study on therapeutic effect between SXDZ-100 and SDZ-II on chronic functional constipation. *Chinese Acupuncture & Moxibustion*.

[13] Chen C.-Y., Ke M.-D., Kuo C.-D., Huang C.-H., Hsueh Y.-H., Chen J.-R. (2013). The Influence of electro-acupuncture stimulation to female constipation patients. *The American Journal of Chinese Medicine*.

[14] Liu S., Peng S., Hou X., Ke M., Chen J. D. Z. (2008). Transcutaneous electroacupuncture improves dyspeptic symptoms and increases high frequency heart rate variability in patients with functional dyspepsia. *Neurogastroenterology and Motility*.

[15] Bharucha A. E., Pemberton J. H., Locke G. R. (2013). American gastroenterological association technical review on constipation. *Gastroenterology*.

[16] Frank L., Kleinman L., Farup C., Taylor L., Miner P. (1999). Psychometric validation of a constipation symptom assessment questionnaire. *Scandinavian Journal of Gastroenterology*.

[17] Marquis P., De La Loge C., Dubois D., McDermott A., Chassany O. (2005). Development and validation of the Patient Assessment of Constipation Quality of Life questionnaire. *Scandinavian Journal of Gastroenterology*.

[18] Diamant N. E., Kamm M. A., Wald A., Whitehead W. E. (1999). AGA technical review on anorectal testing techniques. *Gastroenterology*.

[19] Meunier P. D., Gallavardin D. (1993). Anorectal manometry: the state of the art. *Digestive Diseases*.

[20] Wang L.-J., Wang L.-L. (2011). Randomized controlled study on chronic functional constipation treated with grain-shaped moxibustion and acupuncture. *Zhongguo Zhen Jiu*.

[21] Clarke M. C. C., Chase J. W., Gibb S., Hutson J. M., Southwell B. R. (2009). Improvement of quality of life in children with slow transit constipation after treatment with transcutaneous electrical stimulation. *Journal of Pediatric Surgery*.

[22] Ismail K. A., Chase J., Gibb S., Clarke M., Catto-Smith A. G., Robertson V. J., Hutson J. M., Southwell B. R. (2009). Daily transabdominal electrical stimulation at home increased defecation in children with slow-transit constipation: a pilot study. *Journal of Pediatric Surgery*.

[23] Leong L. C. Y., Yik Y. I., Catto-Smith A. G., Robertson V. J., Hutson J. M., Southwell B. R. (2011). Long-term effects of transabdominal electrical stimulation in treating children with slow-transit constipation. *Journal of Pediatric Surgery*.

[24] Yik Y. I., Ismail K. A., Hutson J. M., Southwell B. R. (2012). Home transcutaneous electrical stimulation to treat children with slow-transit constipation. *Journal of Pediatric Surgery*.

[25] Peng W.-N., Wang L., Liu Z.-S., Guo J., Cai H.-J., Ni J.-N., Duan J.-X., Yang D.-L. (2013). Analysis on follow-up efficacy and safety of slow transit constipation treated with individualized deep puncture at Tianshu (ST 25): a multi-central randomized controlled trial. *Zhongguo Zhen Jiu*.

[26] Wang C. W., He H. B., Li N., Wen Q., Liu Z. S. (2010). Observation on therapeutic effect of electroacupuncture at Tianshu (ST 25) with deep needling technique on functional constipation. *Zhongguo Zhen Jiu*.

[27] Guo L.-K., Zhang C.-X., Guo X.-F. (2011). Acupuncture combined with Chinese herbal medicine plantain and Senna Granule in treatment of functional constipation: a randomized, controlled trial. *Zhong Xi Yi Jie He Xue Bao*.

[28] Zhang C., Guo L., Guo X., Li G., Guo X. (2013). Short and long-term efficacy of combining Fuzhengliqi mixture with acupuncture in treatment of functional constipation. *Journal of Traditional Chinese Medicine*.

[29] Xu X., Pasricha P. J., Sallam H. S., Ma L., Chen J. D. Z. (2008). Clinical significance of quantitative assessment of rectoanal inhibitory reflex (RAIR) in patients with constipation. *Journal of Clinical Gastroenterology*.

[30] Sangwan Y. P., Solla J. A. (1998). Internal anal sphincter: advances and insights. *Diseases of the Colon & Rectum*.

[31] Liu T. T., Chen C. L., Yi C. H. (2008). Anorectal manometry in patients with chronic constipation: a single-center experience. *Hepatogastroenterology*.

[32] Kenefick N. J., Nicholls R. J., Cohen R. G., Kamm M. A. (2002). Permanent sacral nerve stimulation for treatment of idiopathic constipation. *British Journal of Surgery*.

[33] Kamm M. A., Dudding T. C., Melenhorst J., Jarrett M., Wang Z., Buntzen S., Johansson C., Laurberg S., Rosen H., Vaizey C. J., Matzel K., Baeten C. (2010). Sacral nerve stimulation for intractable constipation. *Gut*.

[34] Yin J., Chen J. D. Z. (2010). Gastrointestinal motility disorders and acupuncture. *Autonomic Neuroscience: Basic and Clinical*.

[35] Clarke M. C. C., Chase J. W., Gibb S., Robertson V. J., Catto-Smith A., Hutson J. M., Southwell B. R. (2009). Decreased colonic transit time after transcutaneous interferential electrical stimulation in children with slow transit constipation. *Journal of Pediatric Surgery*.

[36] Clarke M. C. C., Catto-Smith A. G., King S. K., Dinning P. G., Cook I. J., Chase J. W., Gibb S. M., Robertson V. J., Hutson J. M., Southwell B. R. (2012). Transabdominal electrical stimulation increases colonic propagating pressure waves in paediatric slow transit constipation. *Journal of Pediatric Surgery*.

[37] Tomita R., Howard E. R. (2008). Clinical studies on anorectal myectomy for chronically constipated patients with outlet obstruction in childhood. *Hepato-Gastroenterology*.

[38] Ragg J., McDonald R., Hompes R., Jones O. M., Cunningham C., Lindsey I. (2011). Isolated colonic inertia is not usually the cause of chronic constipation. *Colorectal Disease*.

